# Ground Tire Rubber Particles as Substitute for Calcium Carbonate in an EPDM Sealing Compound

**DOI:** 10.3390/polym15092174

**Published:** 2023-05-03

**Authors:** Vanessa Spanheimer, Gamze Gül Jaber, Danka Katrakova-Krüger

**Affiliations:** TH Köln, 51643 Gummersbach, Germany

**Keywords:** end-of-life-tires, ground tire rubber, EPDM, recycling

## Abstract

Ground tire rubber (GTR) is a product obtained by grinding worn tire treads before retreading them or via the cryogenic or ambient temperature milling of end-of-life tires (ELTs). The aim of this study is to evaluate if calcium carbonate can be substituted by GTR and, if so, to what extent. Different types of ground tire rubber are incorporated in an EPDM (ethylene–propylene–diene–rubber) model compound as partial or complete substitutes of calcium carbonate. The raw compounds and the vulcanizates are characterized to identify the limits. In general, it is apparent that increasing amounts of GTR and larger particles degrade the mechanical properties. The GTR also influences the vulcanization kinetics by reducing the scorch time up to 50% and vulcanization time up to nearly 80%. This is significant for production processes. The compounds with one-third substitution with the smaller-particle-size GTR show mostly similar or even better properties than the reference.

## 1. Introduction

The unique viscoelastic properties of rubber are due to chemically crosslinked polymer chains. These crosslinks prevent the polymer chains from sliding off when a force is applied and are responsible for the reversibility of the shape. However, this property is precisely what makes it difficult to recycle. Rubber products cannot be re-melted like thermoplastics or metals due to the crosslinking. Rubber compounds are a complex mixture of polymer, reinforcing (mostly carbon black) and inactive fillers, softeners, crosslinking agents such as sulfur and other chemicals such as processing aids or crosslinking activators and accelerators [1]. Most of these materials are derived from crude oil.

This leads to two current problems: waste management and a large CO_2_ footprint. The amount of end-of-life-tires in 2020 in Germany alone was estimated to be 675,000 t/a; globally, 1 billion tires reach their end of life every year [2,3]. So far, 30% of ELTs are retreaded, especially truck tires, and reused; 50% go into energy recovery, which means they are simply burnt, especially in cement plants; and 20% are recovered as crumb or ground rubber, used in construction for pavements, fall protection on playgrounds, stable mats, sports tracks and more [4,5]. The problem is that cement plants use fewer and fewer tires in their process [6]. So, for a large number of ELTs, this recycling strategy needs to be replaced. Retreading and reusing (truck) tires is limited due to quality, so the increased use of crumb or ground tire rubber is necessary. Therefore, new applications of GTR need to be developed. The GTR can be added in small amounts to other rubber compounds, allowing the compound properties to be maintained. This has already been carried out for different applications. Usually, they are used in addition to or as substitute of the polymer [5,7,8,9]. Most studies use GTR from ELTs, as they are available in high amounts. Most of the time, the use of GTR in new rubber compounds deteriorates the mechanical properties. In particular, the decrease in tensile strength and elongation at break has been reported. In different studies, it was found that there are also some compounds which are not affected negatively by the incorporation of GTR but show even better results. A dependency on the virgin rubber used in the recipe can be seen [10,11,12,13,14,15,16,17,18,19,20,21,22,23,24,25,26,27]. Moreover, the finer the GTR particles are, the better the properties [18,20,28]. In contrast to the tensile properties, the tear strength of different GTR-filled compounds was at least at the same level as the reference or was even improved [11,14,15,16,20,25].

There are also studies that show different approaches to the additional incorporation of GTR in common rubber compounds. They used GTR to create new materials with special properties. These studies show, for example, that GTR can be used in self-healing compounds and have a positive effect on this property [29]. The use of GTR in brakes reduces friction and wear [30]. Using GTR alone with magnetite and a crosslinking system creates a magnetorheological elastomer [31]. The modification of GTR through devulcanization or additives can improve the bonding between the matrix and GTR particles but cannot completely overcome the deterioration of the mechanical properties [5,8,10,11,12,13,14,15,16,18,19,20,21,22,23,24,25,26,27,32].

Almost every company now sets sustainability goals and needs to reduce their emissions and CO_2_ footprint. Producing rubber products is highly energy consuming, and the raw materials derived from crude oil also have a high impact on emissions. Our goal is to use the recycled material, ground tire rubber, in higher amounts in ethylene–propylene–diene–rubber (EPDM) sealing compounds, not on top of but as a substitute for the inactive filler, calcium carbonate, which is a new approach. EPDM sealings are found in different areas such as in automotives, buildings and domestic appliances. Our model compound is an automotive sealing. As the density of GTR is lower than the density of calcium carbonate, we should be able to reduce the rolling resistance, which is important in the automotive field. This leads to lower fuel/energy consumption and an additional emission reduction during the lifetime of an automotive.

## 2. Materials and Methods

### 2.1. Materials

A typical compound for EPDM sealings was used as a reference. Recipes in the rubber industry are given in parts per hundred rubber (phr). This means that the share of every material is related to 100 parts of rubber. The model compound used contained approximately 25 wt% EPDM rubber; 28 wt% carbon black as reinforcing filler; 20 wt% inactive filler, which was calcium carbonate; 22 wt% softener oil; and around 5 wt% chemicals such as a crosslinking system and processing aids. The compounds were crosslinked with sulfur. The amount of calcium carbonate was substituted partially and completely (33% ≙ 25 phr, 66% ≙ 50 phr and 100% ≙ 80 phr; 100% means all of the calcium carbonate was substituted by GTR) with different types of ground tire rubber (see Table 1).

Cryogenic ground tire rubber (CryoGTR) with particle sizes of 400 µm and 200 µm and ambient ground tire rubber (AmbGTR) derived from truck tires from MRH Mülsener Rohstoff- und Handelsgesellschaft were used. In addition, the ambient ground tire rubber was sieved with the sieving machine AS 400 control from Retsch to obtain lower particle sizes of 250 µm, similar to cryogenic ground tire rubber, which has particle sizes of 200 µm. It was not possible to incorporate all GTR particles with 400 µm sizes for both types, ambient and cryogenic, into the rubber compound. Some particles fell off directly, and an even distribution of all particles could not be achieved. Due to these problems during the mixing, the GTR samples with bigger particles could not be used for the complete substitution of calcium carbonate. 

In the following sections, the different compounds with GTR are labeled using the percentage substitution, GTR type and size, for example, 33%-AmbGTR400.

### 2.2. Methods

Microscopic pictures taken with the optical microscope SZX10 (Olympus, Hamburg, Germany) show the morphology of the GTR samples. The particle size distribution was measured with the Mastersizer 3000 (Malvern Panalytical GmbH, Kassel, Germany) via laser diffraction measurements in dry dispersion.

The compounds were mixed on the Polymix 150 L roll mill (Servitec Maschinenservice GmbH, Wustermark, Germany) in two stages—first, mixing the master batch containing all materials except the crosslinking system, and second, mixing the master batch with the crosslinking system. Next, 2 mm and 8 mm thick test slabs were vulcanized at 180 °C and 150 bar according to the curing test data.

The curing tests showed the vulcanization behavior of the compounds. They were performed with the Rubber Process Analyzer RPA Flex from TA Instruments (Eschborn, Germany) at 180 °C for 10 min.

Tensile strength with S2 dumbbell specimens and tear resistance with Graves angle test pieces were measured according to DIN 53504 and DIN ISO 34-1 with the 10 kN allround table top universal testing machine from Zwick Roell (Ulm, Germany). Shore A hardness was measured on 8 mm thick specimens according to DIN ISO 48-4 with a total load of 1 kg, using the hardness tester from Karl Frank GmbH (Weinheim-Birkenau, Germany). The compression set according to DIN ISO 815 was measured at 100 °C 22 h in a Heraeus oven, and rebound elasticity was determined according to DIN 53512 with Rebound Tester 5109 from Zwick. To determine the density according to DIN EN ISO 1183, the specimens were measured via immersion in water with the XS204 Deltarange (Mettler Toledo, Gießen, Germany) scale. All values are given as mean values.

## 3. Results

**Figure 1:**
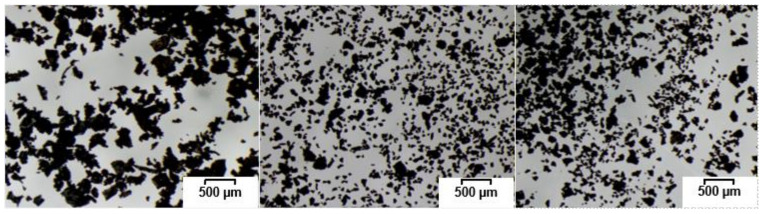
Microscopic pictures of ambient GTR 400 µm (**left**), cryogenic GTR 400 µm (**middle**) and cryogenic GTR 200 µm (**right**).

Figure 1 shows the morphologies of the different GTR samples. The ambient GTR revealed bigger particles with a rough surface due to grinding at room temperature, at which rubber is in its viscoelastic state. In contrast, the cryogenic GTR had smaller particles with sharp edges. As the grinding process took place at temperatures lower than the glass transition temperature of rubber, the rubber behaved and broke like glass. The surface area of the same-sized particles of the ambient GTR was therefore larger than that of the cryogenic GTR, which was much smoother. The D10 (10% of all particles are smaller than this size) and D90 (90% of all particles are smaller than this size) particle size results for the different GTR sample showed the particle size distribution. AmbGTR400 had a D10 of 117 µm and D90 of 462 µm; the sieved AmbGTR250 had a D10 value of 65 µm and a D90 value of 294 µm. The cryogenic GTR samples had a D10 value of 84 µm and a D90 value of 375 µm for CryoGTR400 and a D10 value of 76 µm and a D90 value of 263 µm for CryoGTR200.

The curing tests (see Table 2) showed the changes in the crosslink density. The higher the torque difference (Δ Torque), the higher the crosslink density was, and therefore, other properties such as tensile strength and hardness were higher. With an increasing amount of GTR, the torque difference decreased significantly: around 30% for the partially substituted compounds and nearly 50% for the completely substituted compounds from 15.84 dNm to around 10 dNm and around 8.5 dNm, respectively. At the beginning of vulcanization, the time at which 10% of the highest torque value was reached was measured. This time is called the t_10_ or scorch time. This time was also strongly reduced with an increasing amount of GTR, from 0.9 min to approximately 0.6 min for both 33%-AmbGTR samples, 0.66 min for the 33%-CryoGTR, around 0.47 min for the 66%-AmbGTR and 0.57 min for 66%-CryoGTR, and for complete substitution to 0.43 min for the ambient GTR and 0.48 min for the cryogenic GTR. To measure the time to nearly complete (90%) crosslinking, the t_90_ value was used, which was also used as the optimum curing time. Again, a decrease with an increasing GTR amount could be seen. The reference compound needed 4.74 min for vulcanization. A significant drop was observed for the GTR compounds: up to 77% for the ambient GTR and up to 71% for the cryogenic GTR. As seen with the t_10_, cryogenic GTR in the compound experienced a more minor decrease than the ambient GTR. In contrast to the torque change, the vulcanization times of t_10_ and t_90_ differed significantly between the ambient and cryogenic GTR.

After vulcanization, the mechanical properties were tested. The tensile strength of the reference compound was around 7.7 MPa. The compounds with 33% GTR only had a tensile strength of 4.9 MPa, except 33%-CryoGTR200, with around 5.5 MPa; this already showed significant deterioration. A further increase in GTR led to even lower values of around 4.1 MPa for the 66%-AmbGTR and 3.6 MPa for the 66%-CryoGTR. Finally, the total substitution of calcium carbonate with the finer GTR led to a tensile strength of 2.6 and 2.3 MPa, respectively, which was a drop of nearly 70%. The elongation at break did not differ much from the reference of around 300%, except the 66%-AmbGTR400 compound showed lower elongation at 235%. As the values of tensile strength, being an ultimate property, showed quite high deviations, the stress at 100% elongation and 300% elongation were used for better comparability. The stress at 100% elongation for the reference was 2.5 MPa. With 33% GTR, this value dropped to around 1.8 MPa. Increasing the GTR amount to 66% and 100% decreased the stress at 100% elongation further, except for the 66%-AmbGTR400 compound, which still showed quite a high value compared to the other compounds with 66% substitution. As the elongation at break lay at around 300%, the values of stress at 300% were similar to the tensile strength. Furthermore, some specimens did not reach 300% elongation. This was especially seen in the 66%-CryoGTR400 compound, as there was a value for the stress at 300% elongation, but the mean value for the elongation at break was slightly under 300%. The tear resistance of the compounds with 33% and 66% GTR showed even higher values than the reference. Smaller particle sizes showed higher tear resistances than bigger particles. The compounds with complete substitution showed lower tear resistance than the reference, which indicated that this property is at its maximum between 66% and 100% substitution of GTR.

**Figure 2:**
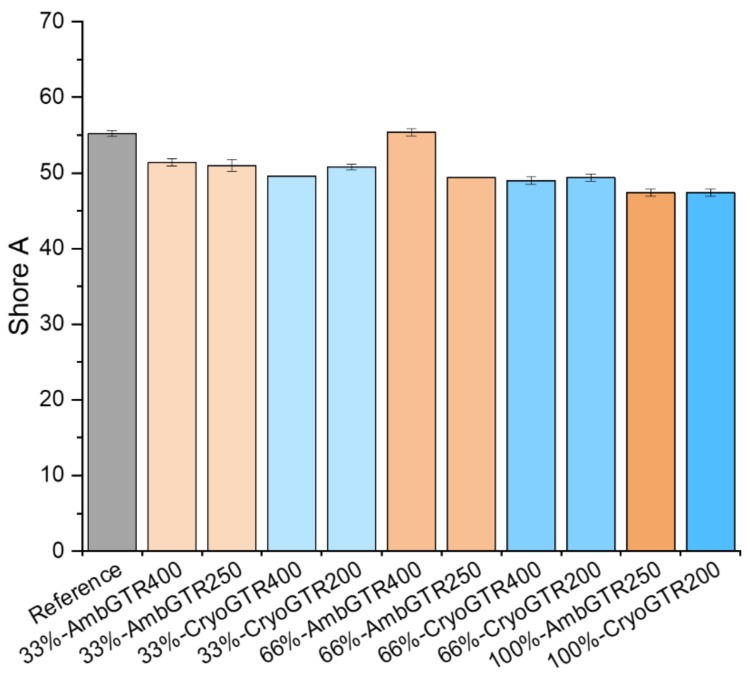
Shore A hardness of all compounds.

The Shore A hardness of the reference was 55. With more GTR, the hardness slightly decreased, except for 66%-AmbGTR, which had the same hardness as the reference (see Figure 2).

The elasticity of the materials could be measured via a compression set and rebound test. The compression set was used to determine the remaining deformation after compression. The lower the compression set, the more suitable the compound was for the sealing applications. The rebound elasticity reflected the percentage value of the recovered energy after the pendulum impact.

**Figure 3:**
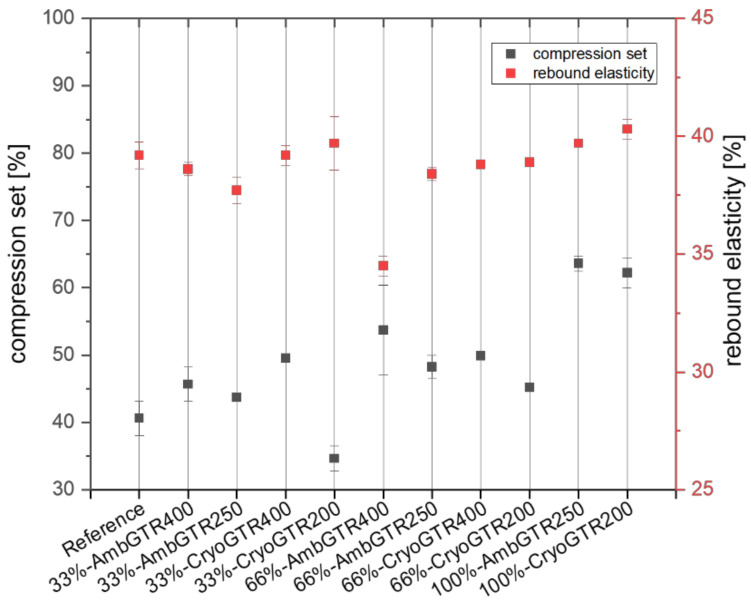
Compression set and rebound elasticity of all compounds.

As the amount of GTR increased, the compression set increased from around 40% (reference) to over 60% for the completely substituted compounds, except for the 33%-CryoGTR200 compound, which was even lower than the reference. The rebound elasticity of the reference was around 40%. All GTR-filled samples—except 66%-AmbGTR400 with around 35%—showed similar rebound elasticity to the reference (see Figure 3).

**Figure 4:**
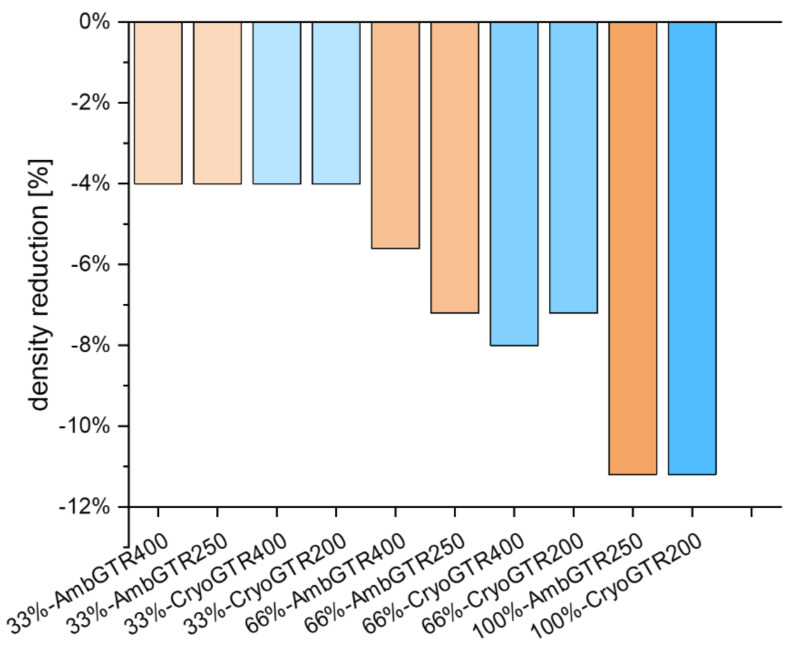
Density reduction in the compounds with GTR.

With a substitution of 33% with GTR, a total compound density reduction of 4% was already achieved. With 66% GTR, a density reduction ranging from 5.6 to 8% could be yielded. The 66% GTR substitution with the finer GTR showed the same density reduction, while the 66% substitution with the more coarse GTR differed. Additional measurements confirmed this finding. A complete substitution with GTR led to a density reduction of 11.2% (see Figure 4).

## 4. Discussion

The microscopic pictures and particle size distribution of the GTR samples showed two main influencing factors on the properties of the rubber compounds—surface area due to the grinding process and particle size distribution. The ambient grinding led to bigger GTR particles with a rough surface, which facilitated the mechanical bonding of the polymer chains with the GTR particles. In contrast, the cryogenic GTR particles had a smoother surface, but the particle size distribution clearly showed that the cryogenic GTR had significantly more finer particles, therefore resulting in a higher surface area than the same amount of ambient GTR. This is in line with the findings of other studies [5,9]. The curing tests revealed that the crosslink density reflected in the torque difference decreased with the increase in the amount of GTR. The minimum torque was elevated because the already-vulcanized GTR particles increased the viscosity [7,10,12,28], and the maximum torque was reduced because the sulfur inside the uncured rubber matrix migrated to the already-cured GTR particles, which led to less crosslinking in the compound [9,28,33]. The particle size distribution and surface area seemed to have no impact; only the amount had an effect. In contrast, the vulcanization times t_10_ and t_90_ showed a dependency on the amount and surface area. The cryogenic GTR compounds had higher t_10_ and t_90_ values than the ambient GTR compounds. A reduction in the processing time of up to 80% could be seen. This could be explained with the migration of unreacted curatives from the GTR into the polymer matrix, which accelerated the vulcanization, as other studies have also shown [5,8,9,10,18]. In contrast, Jacob et al. [28] mixed additional ground EPDM rubber particles into an EPDM compound and found only a marginal decrease in the scorch time. This could have been due to the same composition of the ground EPDM and the matrix, as in this study, the reference compound was vulcanized, ground and mixed into new compounds.

The mechanical properties of the vulcanized compounds were also mainly influenced by the amount of GTR. Again, the tensile strength decreased with increases in the amounts of GTR due to sulfur migration and therefore less crosslinking, as well as the much larger particle sizes of the GTR compared to calcium carbonate, which had particle sizes of around 2 µm. The 66%-AmbGTR400 compound showed a significantly lower elongation at break than the other compounds with the same amount of GTR. This cannot be explained yet. As this compound also noticeably differed in the other tests, it may have either been due to the mixing or to some unknown impurities within the GTR. This trial needs to be carried out again to determine the reason. Depending on the virgin rubber used in the compounds and the amount of GTR, different findings have been presented in the literature. While tensile strength and elongation at break in natural rubber (NR) compounds deteriorated [10,11,12,13,17,19,20,21,22,23,24,25,26], nitrile butadiene rubber (NBR) with incorporated GTR showed improved tensile properties [22,23]. Studies on styrene butadiene rubber (SBR) with GTR showed both tendencies [13,16,18,27]. In EPDM, nearly constant values for tensile strength and elongation at break were measured, which may have been due to the significantly smaller ground rubber particles used, with an average size of about 25 µm [28].

The tear resistance was higher for the partially substituted compounds. This can be explained by the additional amount of carbon black within the GTR particles [5,8], as well as the aforementioned sulfur migration into the GTR particles. However, with further increases in the amount of GTR, there was a decrease in tear resistance, which may have been due to the areas of deficient bonding between the GTR particles and the matrix, which acted as crack initiation points. It is also possible that with increased loading, a percolation level was reached, leading to GRT particles clustering with poor adhesion between them. GTR particles in other rubber compounds also showed improved or at least similar tear strengths to the reference [11,14,15,16,20,25].

The Shore A hardness was slightly lower for the GTR compounds, most probably due to sulfur migration and therefore less crosslinking. Again, 66%-AmbGTR400 was an exception, with a similar hardness to the reference. In other studies, it was found that hardness increased with an increasing amount of GTR, as they found a higher crosslink density and also explained these results by the presence of reinforcing fillers [17,19,20,23,24].

The compression set became higher with the increasing amount of GTR. Again, this could have been related to sulfur migration. Less sulfur in a matrix polymer lead to a shift in the sulfur to accelerators ratio, and therefore, more polysulfidic crosslinks are built. At elevated temperatures, existing polysulfidic crosslinks may cleave and rearrange at other double bonds of the polymer chains. The newly formed crosslinks tend to fix the specimen in the compressed state [34]. This means that a high number of polysulfidic crosslinks can lead to a higher compression set. Otherwise the possible crosslink sites in EPDM (diene) are limited. It is also possible that crosslinks in the GTR particles consisting mostly of unsaturated rubber polymers like NR, BR and SBR and conventionally crosslinked with polysulfidic crosslinks for the needed dynamical properties are changed. As other results indicate, the crosslink density of the GTR-filled compounds was lower. To find the reason behind the increasing compression set with certainty, further studies are needed. The exception of 33%-Cryo-GTR200, which had an even lower compression set than the reference, cannot be explained yet. In terms of rebound elasticity, only 66%-AmbGTR400 differed from the reference.

The density of the compounds could be significantly reduced. This was due to the density difference between calcium carbonate, which was 2.6 g/cm^3^, and GTR, which was around 1.1–1.3 g/cm^3^. Generally, it was possible that the varying densities of the 66% GTR compounds were due to voids within the material. However, at the cutting edges of the specimens and on the surface of the test slabs, no porosity was visible. Another possible reason may have been due to that fact that not all of the GTR was incorporated or evenly distributed during mixing. While mixing the compounds with the bigger GTR particles, the incorporation of these particles into the rubber matrix was still possible but difficult for the 66% substitution, but not for 100%. Probably, 66% is close to the limit. This can explain why the 66%-AmbGTR400 compound only showed a density reduction of 6%, while the 66%-CryoGTR400 compound showed a higher density reduction of about 8%. Moreover, the density reduction in the compounds with 66% substitution with finer ambient and cryogenic GTR was equal. This supports the assumption of poor particle incorporation.

Overall, the 33%-CryoGTR200 compound had the best properties compared to the reference due to the smaller particle sizes, resulting in higher surface area, and the lower amount of GTR.

## 5. Conclusions and Outlook

A 33% replacement of calcium carbonate with GTR can be realized without further recipe adjustments; only the vulcanization process needs to be adjusted as the processing times are reduced up to 65%. Compensation for the sulfur migration is possible by adjusting the crosslinking system. Finer GTR particles are preferred, as they are better to incorporate and exhibit better properties than the GTR with bigger particles. For higher loadings with smaller particles (much closer to the size of the calcium carbonate that is substituted), activation with chemicals and/or the devulcanization of the GTR particles might help to obtain the required properties. The density reduction is already a significant and important benefit that helps to reduce the rolling resistance and therefore increase the mileage. As dispersion measurements of GTR in the rubber matrix are not feasible with methods to determine the dispersion of other fillers such as carbon black or silica, other methods such as indentation or atomic force microscopy may be useful to obtain a better insight [35,36].

Further studies include mixing with an internal mixer, as this has better reproducibility than mixing on a roll mill. The results of this and other studies show that changes occur in crosslinking. This will be considered with accompanying measurements of the crosslink density. Using finer GTR particles as well as ground rubber made from EPDM production waste is expected to be favorable, which is, by now, a challenge for the grinding process and is expensive [37,38]. Currently, EPDM ground rubber is not available on a large scale. Adjustments of the recipes for better incorporation, compensation for the sulfur migration as well as the devulcanization of GTR are also planned [10,32,39]. The results of this study and others—which show quite different influences of GTR on rubber compounds, even on the same virgin rubber—emphasize that for every use case, an individual recipe and processing adjustment is needed.

After these adjustments, a cost analysis considering not only GTR but also the chemicals needed to improve the incorporation and bonding, as well as the process adjustments for the required properties, can be performed to evaluate the economic impact of this approach. Another step is the evaluation of the effect on the CO_2_ balance of the product.

## Figures and Tables

**Table 1:** Recipes of the different compounds with the corresponding percentages of calcium carbonate substitution in brackets.

	Reference	AmbGTR400	AmbGTR250	CryoGTR400	CryoGTR200
Polymer EPDM	100 phr	100 phr	100 phr	100 phr	100 phr
Carbon black	110 phr	110 phr	110 phr	110 phr	110 phr
Calcium carbonate	80 phr	55 phr 30 phr	55 phr 30 phr 0 phr	55 phr 30 phr	55 phr 30 phr 0 phr
Softener oil	85 phr	85 phr	85 phr	85 phr	85 phr
Crosslinking system and additives	18.6 phr	18.6 phr	18.6 phr	18.6 phr	18.6 phr
Ground tire rubber	0 phr	25 phr (≙33%) 50 phr (≙66%)	25 phr (≙33%) 50 phr (≙66%) 80 phr (≙100%)	25 phr (≙33%) 50 phr (≙66%)	25 phr (≙33%) 50 phr (≙66%) 80 phr (≙100%)

**Table 2:** Results of curing tests and mechanical properties of all compounds.

	0% GTR	33% GTR				66% GTR				100% GTR	
	Refer-ence	Amb GTR400	Amb GTR250	Cryo GTR400	Cryo GTR200	Amb GTR400	Amb GTR250	Cryo GTR400	Cryo GTR200	Amb GTR250	Cryo GTR200
Curing tests											
Δ Torque (dNm)	15.84	10.57	9.92	10.26	10.06	10.63	9.47	9.32	9.14	8.37	8.73
t_10_ (min)	0.90	0.60	0.57	0.65	0.66	0.49	0.45	0.57	0.56	0.43	0.48
t_90_ (min)	4.74	1.98	1.77	2.41	2.20	1.37	1.29	1.61	1.66	1.08	1.36
Mechanical properties											
Tensile strength (MPa)	7.70	4.91	4.88	4.84	5.47	4.12	4.17	3.56	3.64	2.57	2.33
Elongation at break (%)	303	300	317	308	340	235	309	293	314	304	272
Stress at 100% elongation (MPa)	2.52	1.82	1.74	1.82	1.86	2.11	1.66	1.53	1.51	1.24	1.25
Stress at 300% elongation (MPa)	7.74	4.93	4.75	4.80	5.04	/	4.14	3.60	3.53	2.59	/
Tear resistance (kN/m)	19.55	23.06	24.01	21.67	23.60	22.36	21.20	20.92	21.56	16.99	14.67

## Data Availability

The data presented in this study are available on request from the corresponding author.
